# Temporal changes within the (bladder) tumor microenvironment that accompany the therapeutic effects of the immunocytokine NHS-IL12

**DOI:** 10.1186/s40425-019-0620-2

**Published:** 2019-06-11

**Authors:** Y. Maurice Morillon, Zhen Su, Jeffrey Schlom, John W. Greiner

**Affiliations:** 1 Laboratory of Tumor Immunology and Biology, Center for Cancer Research, National Cancer Institute, Bethesda, MD USA; 2 EMD Serono, Rockland, MA USA

**Keywords:** Non-muscle invasive bladder cancer, Immunotherapy, NHS-muIL12

## Abstract

**Background:**

While significant strides in the treatment of metastatic bladder cancer have been made with immune checkpoint inhibitors, the treatment of carcinoma in situ and non-muscle invasive, non-metastatic (superficial) human urothelial carcinoma, also termed non-muscle invasive bladder cancer (NMIBC), remains intractable with bacillus Calmette-Guerin (BCG) employed as the standard of care. In this study, an immunocytokine, NHS-muIL12, which consists of two molecules of murine IL-12 fused to NHS76, a tumor necrosis-targeting human IgG1, was examined as an immunotherapeutic in an orthotopic MB49^luc^ bladder tumor model.

**Methods:**

The antitumor activity of systemic administration of NHS-muIL12 was investigated on MB49^luc^ tumors, an aggressive, bioluminescent orthotopic bladder cancer model. Temporal studies were carried out on MB49^luc^ bladder tumors harvested during various time points during NHS-muIL12 treatment and cellular changes associated with the reduction in tumor burden following NHS-muIL12 were determined by flow cytometry. Effects of those changes on the proliferation/activation of lymphoid cells were also determined.

**Results:**

Studies revealed a significant reduction in MB49^luc^ bladder tumor burden occurring between days 3 and 6 after the third and final systemic administration of NHS-muIL12. Temporal analyses of the MB49^luc^ bladder tumor microenvironment (TME) initially revealed a large accumulation of myeloid-derived suppressor cells (MDSCs) and macrophages that elicited potent immunosuppression. Immunosuppression was characterized by the inability of CD4^+^ and CD8^+^ T cells to respond to broad-based immune stimulants. NHS-muIL12 administration resulted in temporal-dependent reductions in the number of MDSCs, macrophages and tumor-associated TGF**-**β, which culminated in a re-ignition of CD4^+^ and CD8^+^ T cells to elicit potent antitumor responses against MB49^luc^ bladder tumors.

**Conclusions:**

These findings provide strong evidence that the systemic administration of an immunocytokine consisting of a tumor-targeting Ig through recognition of DNA and DNA-histone complexes coupled to muIL-12 can effectively target the bladder TME; this significantly reduces the myeloid cellular compartment and reverts an immunosuppressive to an immunopermissive TME, ultimately resulting in antitumor effects. These studies provide further rationale for the employment of NHS-IL12 as an immunomodulator and clinical immunotherapeutic for NMIBC.

## Introduction

Bladder cancer is the fifth most common cancer in the United States [1] and, until recently, few new treatment approaches had emerged. Coupling an understanding of mutational heterogeneity underlying the influx of immune cells with the suppressive actions of the PD-1/PD-L1 axis has led to the development and regulatory approval of immune cell checkpoint inhibitors, including avelumab (PD-L1), atezolizumab (PD-L1), durvalumab (PD-L1), pembrolizumab (PD-1), and nivolumab (PD-1), for the treatment of metastatic bladder cancer [2–6]. However, since the pioneering work of Morales et al. in 1976 [7], the standard of care for carcinoma in situ and non-muscle invasive, non-metastatic urothelial carcinoma remains the intravesical instillation of attenuated bacillus Calmette-Guerin (BCG). The mechanism of BCG action remains elusive, yet most investigators believe that the influx of immune cells is a crucial component [8]. Approximately 30–50% of patients do not respond initially to BCG or relapse within 5 years of treatment [8, 9]. This limited effectiveness of BCG has underscored the need for additional therapies to reduce recurrence and improve survival for both BCG responders and non-responders.

IL-12 has been an intriguing cytokine due to its powerful proinflammatory effects that include proliferation of activated T cells and natural killer (NK) cells that promote potent T_H1_ cell-mediated immunity [10–13]. Preclinically, recombinant IL-12 (rIL-12) has remarkable antitumor effects against a wide range of malignancies, and has been linked to the generation of a strong antigen-specific immunological response and memory [14, 15]. Translation of those potent T_H1_ properties into successful patient treatment has been stymied by dose-limiting adverse events attributed to the systemic administration of rIL-12 [16]. Consequently, researchers have sought to develop novel approaches to selectively target IL-12 that might mitigate the systemic toxicities while taking advantage of its antitumor actions [17–21].

In previous studies [17, 22], instillation of murine rIL-12 admixed with chitosan, a bioadhesive, significantly inhibited the growth of orthotopic MB49^luc^ bladder tumors. Successful antitumor efficacy directed at the MB49^luc^ bladder tumors was lost when either CD4^+^ or CD8^+^ T cells were absent, indicating the requirement of an intact host immune system. In the present study, murine IL-12 was administered as part of a fusion protein engineered by genetically fusing two murine IL-12 heterodimers to the C-termini of the heavy chains of the NHS76 antibody. NHS76 is a fully human, phage display-derived IgG1 antibody that recognizes DNA-histone epitopes that are exposed within the necrotic regions of tumors [23, 24]. In contrast to BCG, which is administered intravesically, NHS-Il12 is administered systemically. Previous studies highlight the advantages of delivering IL-12 as a fusion protein or “immunocytokine” over its administration as a recombinant protein [18, 23, 25]. Advantages include: (a) significant reduction of bioactivity (> 90%), i.e., less potential toxicity, by coupling murine IL-12 to the NHS76 antibody, (b) a sustained in vivo pharmacokinetic profile of NHS-muIL12, and (c) equal or superior antitumor activity when compared with rIL-12 in different types of murine tumors. The structure of NHS-IL12 has been previously reported [18]. Targeting of NHS-muIL12 through tumor necrosis–TNT antibody recognition should mitigate much of the systemic toxicity of rIL-12 administration, while delivering the potent T_H1_ cytokine to the tumor microenvironment (TME). While the robust anti-tumor properties of NHS-IL12 have been described [18], the present study examined the cellular events that accompanied antitumor efficacy within the MB49^luc^ bladder TME following systemic administration of NHS-muIL12. In short, antitumor effects of NHS-muIL12 are associated with treatment-related reductions in myeloid derived suppressor cells (MDSCs) and macrophages and a return to an immunopermissive TME, which ultimately permits efficient tumor clearance via T cell–mediated killing.

## Materials and methods

### Animals and cell lines

Female C57BL/6 mice were purchased from The Jackson Laboratory or Charles River Laboratories and housed in microisolator cages in pathogen-free conditions. Mice used for in vivo antitumor studies were 16 to 18 weeks old at the start of study. Animal care followed The Guide for Care and Use of Laboratory Animals (National Research Council). MB49 parental (murine transitional bladder carcinoma) and MB49 LucSH^+^ cells (MB49^luc^) were grown and maintained as previously described [17].

### Murine tumor models

Intravesical instillation of orthotopic MB49^luc^ bladder tumors was carried out as previously described [17]. Tumor take was confirmed by in vivo imaging 7–10 days later, at which time mice were placed into appropriate treatment groups with equal tumor burden.

### Bioluminescent imaging

Orthotopic bladder tumor growth was detected via IVIS Lumina In Vivo Imaging (Caliper Life Sciences, Alameda, CA). The abdominal regions of mice were shaved followed by intraperitoneal (i.p.) injection of 15 mg/kg luciferin salt 10 min prior to imaging. Anesthesia was administered by Forane (Isoflurane, USP, Baxter, Deerfield, IL) inhalation at 2% for the duration of imaging. Bioluminescence signal was read as total flux radiance (photons/sec/cm^2^/steradian) using the Living Image Software, Version 4.1 (Caliper Life Sciences, Hanover, MD).

### Treatments

NHS-muIL12, an immunocytokine consisting of a human IgG1 NHS-76 antibody and two murine IL-12 molecules, was kindly provided by EMD Serono under a Collaborative Research and Development Agreement (CRADA) with the NCI. NHS-muIL12 was stored at +4C and diluted in Dulbecco’s phosphate buffered saline (DPBS) prior to injection [18]. For anti-tumor studies, NHS-muIL12, a control human IgG1 or diluent was injected subcutaneously (s.c.) into the inner thigh. Mice were treated s.c., three times, 3 days apart. That treatment schedule allows for the delivery of biologically active NHS-muIL12 (i.e., IFN-γ production) prior to the development of neutralizing murine anti­human Ig directed against the immunocytokine [18].

### In vitro immune suppression assay

CD45^+^ cells were isolated from MB49^luc^ bladder tumor homogenates of control Ig– or NHS-muIL12–treated animals at 5 days post–final NHS-muIL12 treatment. CD45^+^ cells were isolated via magnetic-activated cell sorting (MACS) separation using Miltenyi CD45+ Microbeads (Auburn, CA). T cells were isolated from the spleens of naïve C57BL/6 mice using the Miltenyi Pan T Cell Isolation Kit according to the manufacturer’s specifications. Isolated cells were incubated at ratios of CD45^+^ tumor-derived cells to T cells ranging from 1:2 to 1:128. Cells were incubated in the presence of soluble αCD3 (clone 145-2C11, eBioscience/Thermo Fisher, Waltham, MA) and αCD28 (clone 27.51, eBioscience), (0.5 and 1.0 μg/ml) for 2 days, after which cell proliferation was determined via Ki67 staining of live/blasting T cells using flow cytometry.

### Flow cytometry analysis

Antibodies used for flow cytometry were purchased from BD Biosciences (San Jose, CA), eBioscience/Thermo Fisher, or BioLegend (San Diego, CA). Fluorescently conjugated antibodies specific for CD3 (145-2C11), CD4 (RM4–5), CD8 (53–6.7), FoxP3 (FJK-16 s), F4/80 (BM8), GR1 (RB6-8C5), CD11b (M1/70), CD44 (IM7), CD38 (90), Ly6G (1A8), Ly6C (AL-21), IFN-γ (XMG1.2), and Ki67 (B56) were used for flow cytometry. Staining and cell counting was performed as previously described [26]. For the detection of intracellular IFN-γ, prior to staining/fixing, single cell suspensions were cultured for 5 h with Cell Stimulation Cocktail (eBioscience/Thermo Fisher); Golgi Plug/Brefeldin A (BD Bioscience) was added for the final 4.5 h of culturing. Cytometry data were acquired utilizing a three-laser FACSVerse (BD Biosciences). Data were analyzed using FlowJo (FlowJo, LLC, Ashland, OR).

### Tumor cytokine detection

MB49^luc^ bladder tumors were collected from mice on day 6 post–final NHS-muIL12 treatment. Tumors were homogenized via mechanical dissociation and centrifuged at 500×g for 10 min. Supernatants were removed, and tumors individually resuspended in EL-Lysis buffer containing Protease Inhibitors (Ray Biotech, Norcross, GA), according to the manufacturer’s recommendations. Levels of IFN-γ and TGF-β were measured in tumor lysates utilizing R&D Systems Quantikine ELISA Kits (Minneapolis, MN).

### Statistical analysis

GraphPad Prism software (GraphPad Prism 7 for Windows, GraphPad Software, Inc., La Jolla, CA) was used to perform all statistical analyses. Details of appropriate statistical analyses are found within each figure legend. Differences were significant when the *P* value was ≤0.05.

## Results

### Temporal antitumor effects of NHS-muIL12 in the MB49^luc^ bladder tumor model

**Fig. 1:**
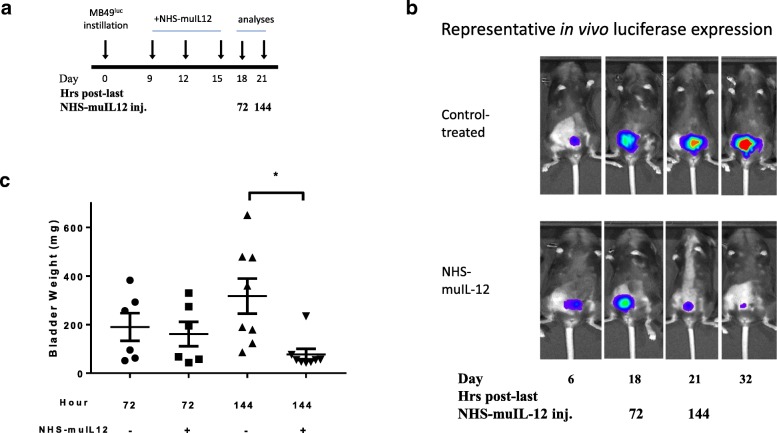
Antitumor effects of NHS-muIL12 on MB49^luc^ bladder tumors. **a** NHS-muIL12 treatment (days 9, 12 and 15) and analyses (days 18, 21) schedule of mice bearing MB49^luc^ bladder tumors. **b** Representative in vivo luciferase expression images taken immediately prior to euthanasia at days 6, 18, 21, and 32 for control Ig– (top row) and NHS-muIL12–treated mice (bottom row). **c** Individual bladder weights from control Ig–treated (circles, triangles) and NHS-muIL12–treated (squares, inverted triangles) mice at 72 and 144 h post–final NHS-muIL12 injection (days 18 and 21). Horizontal lines represent the average bladder weight; error bars represent mean ± SEM, Student’s t-test; *P < 0.05. Data are from a representative experiment that was repeated with similar results

A previous study [27] reported a dose-dependent reduction of MB49^luc^ bladder tumor growth in mice treated with 0.05 to 0.4 μg × 3 NHS-muIL12 with 0.4 μg NHS-muIL12 resulting in complete tumor regression in most mice. Of interest was to examine the cellular changes occurring within the MB49^luc^ bladder TME during the time interval commensurate with the reductions in tumor growth following NHS-muIL12 treatment. In the present study, mice bearing MB49^luc^ bladder tumors were treated with 0.4 μg NHS-muIL12 on days 9, 12 and 15 (Fig. 1a). Intra-vital imaging (Fig. 1b) and individual bladder weights (Fig. 1c) showed active MB49^luc^ bladder tumor growth suppression between days 18 and 21 post-tumor instillation, or 72 and 144 h after the final NHS-muIL12 treatment. At the 72-h time point, there were no discernable differences in MB49^luc^ bladder tumor burden between the control Ig– and NHS-muIL12–treated mice; luciferase-based images were similar (Fig. 1b) as were the bladder weights (i.e., control Ig, 190.2 ± 65.7; NHS-muIL12, 161.1 ± 50.2 mg) (Fig. 1c). At the 144-h time point, the average MB49^luc^ bladder tumor weight from control Ig–treated mice was 312.4 ± 72.1 mg, indicating ongoing MB49^luc^ tumor growth, while those from NHS-muIL12–treated mice were 87.6 ± 22.9 mg, indicating an ongoing treatment-related antitumor response (Fig. 1c). Thus, changes within the MB49^luc^ bladder TME at 72 and 144 h after the final NHS-muIL12 treatment seemed to contribute to the potent antitumor response and became the focus of subsequent study.

### Cellular changes in the MB49^luc^ bladder TME during NHS-muIL12 immunotherapy

**Fig. 2:**
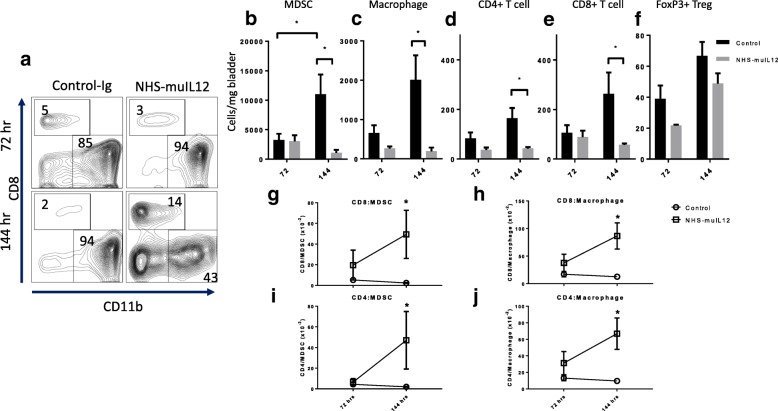
Cellular changes in the MB49^luc^ bladder TME of control Ig– and NHS-muIL12– treated mice at 72 and 144 h after the final NHS-muIL12 treatment. **a** Representative FACS plots of cytotoxic T lymphocytes (CD8, y-axis) vs myeloid (CD11b, x-axis) cells from control Ig– (left column) and NHS-muIL12–treated (right column) mice bearing MB49^luc^ bladder tumors. Plots are representative of live CD45^+^ tumor-derived cells at 72 (top row) and 144 h (bottom row) after the final NHS-muIL12 treatment. Numbers within each quadrant represent percent of total cells. In panels **b-f**, mice bearing MB49^luc^ bladder tumors and treated with control Ig– (black bars) or NHS-muIL12 (grey bars) were euthanized at 72 and 144 h after the final NHS-muIL12 treatment and the lymphoid/myeloid cellular components within the TME were examined by flow cytometry. Absolute number of each cell type/mg bladder weight for (**b**) MDSCs: CD11b^+^Gr1^+^F4/80^−^ (*n* = 9), (**c**) macrophages: CD11b^+^Gr1^−^F4/80^+^ (*n* = 9), (**d**) CD4^+^ T cells (*n* = 9), (**e**) CD8^+^ T cells (*n* = 9), and (**f**) regulatory T cells (Tregs): CD3^+^CD4^+^FoxP3^+^ (*n* = 3) are shown. Panels **g-j** represent ratios of (**g**) CD8^+^/MDSCs, (**h**) CD8^+^/macrophages, (**i**) CD4^+^/MDSCs, and (**j**) CD4^+^/macrophages at 72 and 144 h after the final NHS-muIL12 treatment (*n* = 9). Circles and squares represent control Ig–treated and NHS-muIL12–treated mice, respectively. Error bars (panels b-j) represent mean + SEM, Student’s t-test; *P < 0.05. Data are from a representative experiment that was repeated twice with similar results

Flow cytometry assessed both the frequencies and number of myeloid (MDSCs and macrophages) and lymphoid (i.e., CD4^+^, CD8^+^, CD4^+^FoxP3^+^) cells/mg MB49^luc^ bladder weight at 72 and 144 h after the final NHS-muIL12 treatment. At the 72-h time point, representative FACS plots of the MB49^luc^ bladder TME from control Ig– and NHS-muIL12–treated mice were similar with few (i.e., 3–5%) CD45^+^ CD8^+^ T cells, but a preponderance (i.e., > 85%) of the cells expressing CD11b^+^, a myeloid phenotype (Fig. 2a, top row). Commensurate with the ongoing antitumor effects of NHS-muIL12, at the 144-h time point, the percentage of CD8^+^ T cells was 14% in the TME of NHS-muIL12–treated mice compared with 2.0% in the TME from control Ig–treated mice. The percentage of CD11b^+^ myeloid cells fell from 94% in the control Ig–treated mice to 43% in the MB49^luc^ bladder TME from NHS-muIL12–treated mice (Fig. 2a, bottom row). When the number of MDSCs (Gr1^+^ CD11b^+^ F4/80^−^), macrophages (Gr1^−^ CD11b^+^ F4/80^+^) and CD4^+^, CD8^+^, CD4^+^FoxP3^+^ lymphoid cells were analyzed per mg bladder (Fig. 2b-f), more dramatic changes in both control Ig– and NHS-muIL12–treated mice emerged at the 72- and 144-h time points. In control Ig–treated mice, those changes included: (a) a 3-fold increase in MDSCs (*p* < 0.01, Fig. 2b) and macrophages (*p* = 0.057, Fig. 2c) and (b) trending higher numbers (*p* = 0.1) of CD4^+^, CD8^+^ and CD4^+^FoxP3^+^ T cells (Fig. 2d-f). In contrast, with NHS-muIL12 treatment, the accumulation of MDSCs and macrophages in the MB49^luc^ bladder TME were blunted so that at the 144-h time point the numbers of both MDSCs and macrophages/mg bladder were significantly (*p* < 0.01) lower than those of the control Ig–treated mice (Fig. 2b, c). Similar trends were observed in the T cell compartment, where an increase in both CD4^+^ (Fig. 2d) and CD8^+^ T cells (Fig. 2e) was observed in control Ig–treated mice from 72 to 144 h. The number of CD4^+^ FoxP3^+^ regulatory T cells/mg MB49^luc^ bladder trended higher (not significant) in both the control Ig– and NHS-muIL12–treated mice (Fig. 2f) during the 72- and 144-h time interval. Dendritic (CD11c^+^) and NK (NK1.1^+^) cells were rare, but present in equal numbers, in MB49^luc^ bladder TME from both control Ig– and NHS-muIL12–treated mice (data not shown).

To further examine the dynamic cellular changes in the MB49^luc^ bladder TME that accompanied NHS-muIL12 treatment, the changes in the ratios of lymphoid (CD4^+^ and CD8^+^ T cells) to myeloid (MDSCs and macrophages) cells were determined at the 72- and 144-h time points (Fig. 2g-j). Despite the differences in the number of CD4^+^, CD8^+^, MDSCs and macrophages per mg bladder weight (Fig. 2b-e) at 72 h post–NHS-muIL12 treatment, the ratios of CD8^+^/MDSC (Fig. 2g), CD8^+^/macrophages (Fig. 2h), CD4^+^/MDSC (Fig. 2i), and CD4^+^/macrophages (Fig. 2j) in the MB49^luc^ bladder TME of control Ig– and NHS-muIL12–treated mice were remarkably similar. By 144 h, however, those ratios in mice bearing MB49^luc^ bladder tumors and treated with NHS-muIL12 were significantly higher than for control Ig–treated mice. For example, because of the ongoing accumulation of MDSCs (Fig. 2b) or macrophages (Fig. 2c) in the MB49^luc^ bladder TME of control Ig–treated mice, the CD8^+^:MDSCs/macrophage ratios fell (Fig. 2g, h), as did the CD4^+^:MDSCs/macrophage ratios (Fig. 2i, j). In contrast, CD4^+^/8^+^:MDSC and CD4^+^/8^+^:macrophage ratios were all significantly (*p* < 0.05; vs. control Ig–treated mice) increased in MB49^luc^ bladder TME of NHS-muIL12–treated mice at the 144-h time point (Fig. 2g-j, squares).

### Additional phenotypic changes within the MB49^luc^ bladder TME following NHS-muIL12 treatment

**Fig. 3:**
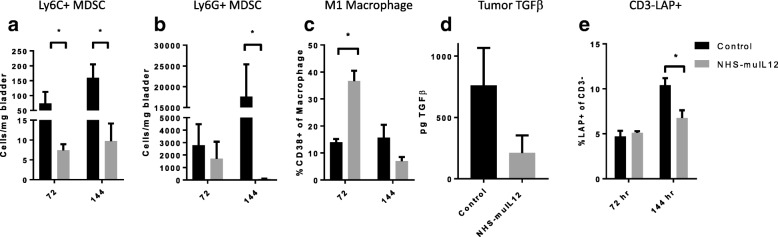
Temporal-dependent phenotypic (72 and 144 h after the final NHS-muIL12 treatment) changes in myeloid/lymphoid cell profiles in the MB49^luc^ bladder TME of control Ig– (black bars) and NHS-muIL12–treated (grey bars) mice. Panels **a**-**c** represent differences in absolute number of cells/mg bladder weight of (**a**) Ly6C^+^ MDSCs, (**b**) Ly6G^+^ MDSCs and (**c**) in the frequencies of M1 of CD11b^+^Gr1^−^F4/80^+^ macrophages. (**d**) Total tumor TGF-β from control Ig– (black bars) and NHS-muIL12–treated (grey bars) mice at the 144-h time point (*n* = 5). **e** Frequencies of LAP^+^ CD3^−^ cells. Error bars represent mean + SEM, Student’s t-test; *P < 0.05, unless otherwise indicated, *n* = 3. Data are from a representative experiment that was repeated twice with similar results

Gr1^+^ CD11b^+^ F4/80^−^ MDSCs can be further divided into Ly6C^+^ monocytic and Ly6G^+^ granulocytic populations and depending on their contextual expression (e.g., tumor microenvironment, site of chronic inflammation) each can evoke immunosuppression via arginase I (Arg I) and inducible nitric oxide synthase (iNOS), thus inhibiting T cell activation [28, 29]. In mice bearing MB49^luc^ bladder tumors and treated with NHS-muIL12, the significant reduction of CD11b^+^ Gr-1^+^ MDSCs comprised both the Ly6C^+^ and Ly6G^+^ populations (Fig. 3a, b); the reduction in the CD11b^+^ Gr-1^+^ Ly6G^+^ cells (Fig. 3b) was more pronounced than that for the monocytic CD11b^+^ Gr-1^+^ Ly6C^+^ MDSCs (Fig. 3a).

The M1 polarization of F4/80^+^ macrophages was determined via staining with CD38 [30]. This inflammatory macrophage population was highest in MB49^luc^ bladder tumors of NHS-muIL12–treated mice at 72 h, followed by a sharp decline by 144 h post–final NHS-muIL12 treatment (Fig. 3c). Early upregulation of the immunopermissive M1 macrophage population may support the NHS-muIL12–mediated antitumor effects via an increase in the overall proinflammatory milieu.

At the study end, 6 days after the final NHS-muIL12 treatment, MB49^luc^ bladder tumors from control Ig– and NHS-muIL12–treated mice were isolated, lysates prepared and total IFN-γ, IL-12 and TGF-β quantitated. No differences were evident in either total IFN-γ or IL-12 levels in bladder tumor lysates from control Ig– and NHS-muIL12–treated mice (data not shown). In contrast, TGF-β levels in the MB49^luc^ bladder tumor lysates from NHS-muIL12–treated mice were reduced by approximately 60% when compared with levels in bladders of control Ig–treated mice (Fig. 3d). Consistent with reduced TGF-β activation [31], expression of the latency-associated peptide (LAP) in the non-lymphoid cell population (i.e., CD3^−^ LAP^+^, which is the N-terminal propeptide of TGF-β) was significantly (*p* < 0.05) reduced at the 144-h time point within the MB49^luc^ bladder TME of NHS-muIL12–treated vs. control Ig–treated mice (Fig. 3e).

### Functional immune suppression within the MB49^luc^ bladder TME

**Fig. 4:**
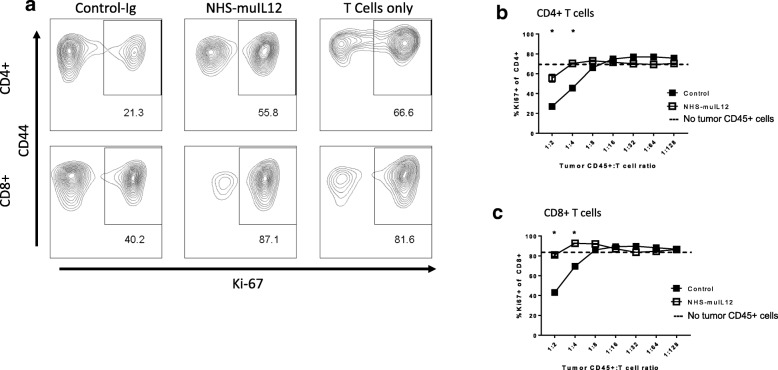
Reversal of immunosuppression within the MB49^luc^ bladder TME following NHS-muIL12 treatment. MB49^luc^ bladder tumors (*n* = 3) from control Ig– and NHS-muIL12–treated mice were isolated 5 days post–final NHS-muIL12 treatment (day 20, see Fig. 1, panel a). CD45^+^ cells were purified from tumor homogenates and cocultured with CD4^+^ and CD8^+^ T cells (effectors) purified from the spleen of C57BL/6 mice in the presence of soluble αCD3 and αCD28 for 48 h as described in Methods. Following in vitro stimulation, cells were stained for T cell (CD3^+^, CD4^+^, CD8^+^), activation (CD44) and proliferative (Ki-67) markers. **a** Representative FACS plots showing the CD44 and Ki67-expressing CD4^+^ (top row) and CD8^+^ (bottom row) T cells after co-incubation with CD45^+^ tumor-derived cells (ratio 1:2) from MB49^luc^ bladder from control Ig– and NHS-muIL12–treated mice. FACS plots indicated as “T cells only” were from cultures containing indicated splenic CD4^+^ or CD8^+^ T cells from naïve B6 mice (i.e., no addition of bladder tumor-derived CD45^+^ cells). Numbers within each FACS plot are the percentage of CD4^+^ and CD8^+^ T cells co-expressing CD44 and Ki67. **b, c** Frequency of CD4^+^ (**b**) and CD8^+^ (**c**) T cell proliferation reported as a percentage of Ki67-expressing cells using CD45^+^ cells from tumors of control Ig– (solid squares) and NHS-muIL12–treated mice (open squares) at ratios CD45^+^ tumor-derived cells to T cells of 1:2 to 1:128. Dashed line in **b** and **c** represents the average proliferation as measured by Ki67-expressing T cells in the absence of tumor-derived CD45^+^ cells. Student’s t-test; *P < 0.05 (vs. control Ig–treated mice). Data are from a single experiment

High numbers of MDSCs and macrophages in the MB49^luc^ bladder TME of control Ig–treated mice would argue for a local immunosuppressive environment that could be reversed by treating with tumor targeting NHS-muIL12. To test that hypothesis, CD45^+^ cells were isolated from MB49^luc^ bladder tumors of control Ig– or NHS-muIL12–treated mice on day 5 after the final NHS-muIL12 treatment. Those CD45-expressing cells were co-incubated with effector splenic T cells from naïve C57BL/6 mice and CD4^+^ and CD8^+^ T cell proliferative response to CD3/28 stimulation was measured via Ki67-expression. Consistent with an active cell-mediated immunosuppressive TME, co-culturing CD45^+^ cells from MB49^luc^ bladder tumors from control Ig–treated mice with splenic effector cells reduced the percentage of Ki67-expressing splenic CD4^+^ and CD8^+^ cells in response to αCD3/28 to 21.3 and 40.2%, respectively (Fig. 4a, left panels). In contrast, no such reduction in the CD4^+^ and CD8^+^ T cell proliferative response to αCD3/28 was observed with the addition of CD45^+^ cells from the MB49^luc^ bladders of NHS-muIL12–treated mice, suggesting little or no cell-mediated immunosuppression (Fig. 4a, middle panels). As a positive control, in the absence of bladder tumor isolated CD45^+^ cells, the percentage of splenic CD4^+^ and CD8^+^ T cells from naïve mice that expressed Ki67 following αCD3/28 in vitro stimulation was 66.6 and 81.6%, respectively (Fig. 4a, right panels: labeled T cells only). Suppression of CD4^+^ (Fig. 4b) and CD8^+^ T cells (Fig. 4c) in response to αCD3/28 stimulation was dependent on the number of CD45^+^ cells isolated from MB49^luc^ bladder tumors and added to the suppression assay. Co-incubation of CD45^+^ cells isolated from MB49^luc^ bladder tumors of control Ig–treated mice with either CD4^+^ or CD8^+^ T cells from naïve mice (ratios of 1:2 and 1:4) significantly reduced the percentage of CD4^+^ or CD8^+^ T cells expressing Ki67 (Fig. 4b, c). That suppression of CD4^+^ or CD8^+^ T cells was either significantly reduced (Fig. 4b) or eliminated (Fig. 4c) when utilizing CD45^+^ cells isolated from NHS-muIL12–treated tumors, suggesting a reversal of immune-cell suppression within the MB49^luc^ bladder TME. Hashed lines represent effector T cell proliferation in the absence of tumor-derived CD45^+^ T cells.

### Immune cell activation in the MB49^luc^ bladder TME following NHS-muIL12 treatment

**Fig. 5:**
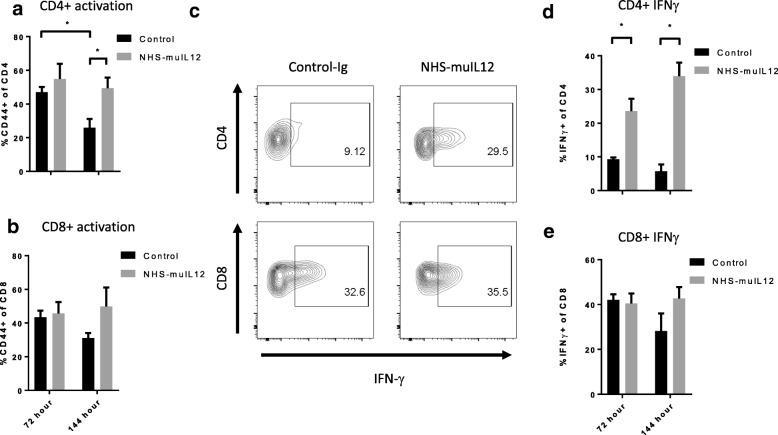
NHS-muIL12 treatment increases T cell activation (CD44 expression) and pro-inflammatory events (i.e., intracellular IFN-γ expression) within the MB49^luc^ bladder TME. **a, b** MB49^luc^ bladder tumors (*n* = 3) from control Ig– (black bars) and NHS-muIL12–treated (grey bars) mice were isolated at 72 and 144 h post–final NHS-muIL12 treatment and single cell suspensions were prepared (see Methods) and stained for CD4^+^, CD8^+^ T cells along with the CD44 activation marker. **c** Representative FACS plots for intracellular IFN-γ staining of CD4^+^ and CD8^+^ T cells after a 5-h in vitro stimulation (see Methods). **d, e** Frequencies of CD4^+^ and CD8^+^ T cells expressing intracellular IFN-γ from MB49^luc^ bladder tumors from control Ig– (black bars) and NHS-muIL12–treated (grey bars) mice at 72 and 144 h post–final NHS-muIL12 administration. Error bars (panels **a, b, d, e**) represent mean + SEM. Student’s t-test; *P < 0.05 (vs. control-treated mice)

Significant reduction in the number of myeloid cells within the TME coincided with growth suppression of MB49^luc^ bladder tumors (Fig. 1c). Furthermore, previous findings established that CD4^+^ and CD8^+^ T cells were both required for the antitumor effects of NHS-muIL12 treatment of mice bearing MB49^luc^ bladder tumors [27]. Those observations argued that the decrease in the number of myeloid cells might be tied to changes in the suppression/activation status of CD4^+^ and CD8^+^ T cells within the bladder TME. To examine that relationship, the activation status of CD4^+^ and CD8^+^ T cells within the MB49^luc^ bladder TME from control Ig– and NHS-muIL12–treated mice were examined. In the MB49^luc^ bladder TME from control Ig–treated mice, the percentage of CD4^+^ and CD8^+^ T cells expressing CD44, a T cell activation marker, dropped from 47 to 43% at 72 h to 26 and 31% at 144 h, respectively, suggesting ongoing/increasing suppression of both immune cell subsets (Fig. 5a, b). No such drop in CD4^+^ and CD8^+^ T cells expressing CD44 was found in the MB49^luc^ bladder TME from NHS-muIL12–treated mice (Fig. 5a, b). Figure 5c shows a representative FACS analysis of intracellular IFN-γ expression by CD4^+^ and CD8^+^ T cells, a marker for a T_H1_ response and CTL precursor cells, isolated from MB49^luc^ bladder tumors from control Ig– and NHS-muIL12–treated mice. The top row illustrates an increased percentage of CD4^+^ T cells that stained positive for intracellular IFN-γ production in the MB49^luc^ bladder TME of NHS-muIL12–treated (i.e., 29.5%) versus control Ig–treated (i.e., 9.12%) mice (Fig. 5c). No such change was found within the CD8^+^ T cell population in the TME of NHS-muIL12–treated mice (Fig. 5c, bottom row). At 72 and 144 h after the final NHS-muIL12 treatment, cytometric analyses revealed significant increases in the percentage of CD4^+^ / IFN-γ^+^ T cells in the MB49^luc^ bladder TME of NHS-muIL12 versus control Ig–treated mice (Fig. 5d). No such increase was found in the CD8^+^ T cell subset (Fig. 5e). Consistent with heightened immunosuppression at the 72- and 144-h time points, a trending reduction of CD8^+^/IFN-γ^+^ T cells was present in the MB49^luc^ bladder TME of control Ig–treated mice (Fig. 5e).

## Discussion

Efforts to identify novel approaches to improve upon the use of BCG as the standard of care for carcinoma in situ and non-muscle invasive, non-metastatic urothelial carcinoma have relied upon a syngeneic murine model of orthotopic instillation of MB49 transitional bladder tumor cells [17, 20]. Known characteristics of the MB49^luc^ bladder tumor model include early tumor necrosis and an immunosuppressive (T_H2_ polarized) TME governed by IL-10 signaling [32–34]. In the present study, the immunosuppressive MB49^luc^ bladder TME was further characterized by the presence of high numbers of myeloid-derived cells, primarily CD11b^+^Gr1^+^F4/80^−^ MDSCs and CD11b^+^Gr1^−^F4/80^+^ macrophages as well as functional suppression of CD4^+^ and CD8^+^ T cell activation that underscored the inability of resident CD8^+^ T cells to control MB49^luc^ bladder tumor growth. These data highlight the importance of the collection of biospecimens and evaluation of immunologic correlates, with particular emphasis on the analyses of MDSCs in patients with urothelial carcinoma [35, 36]. Systemic administration of NHS-muIL12, an immunocytokine that targets IL-12 to areas of tumor necrosis through recognition of exposed DNA-histone epitopes, proved to be a highly efficient immunotherapeutic in mice bearing orthotopic MB49^luc^ bladder tumors.

While the anti-tumor efficacy conferred by NHS-muIL12 has been previously reported [18, 27], mechanistic characterization is still lacking. To address this, a temporal study was designed to identify those changes occurring within the MB49^luc^ bladder TME during the time interval of maximum tumor regression, i.e., at 72 and 144 h after the third and final NHS-muIL12 administration (Fig. 1b, c). The findings revealed excellent concordance between the phenotypic and functional changes within the MB49^luc^ bladder TME and the antitumor responses to systemic NHS-muIL12 administration. At 72 h post–third and final NHS-muIL12 treatment, the tumors still contained a preponderance of CD11b^+^ Gr1^+^ F4/80^−^ MDSCs and CD11b^+^Gr1^−^F4/80^+^ macrophages when compared with CD4^+^ and CD8^+^ T cells, arguing for an ongoing immunosuppressive TME (Fig. 2b, c). At the 72-h post–final NHS-muIL12 treatment, there was no discernable reduction in MB49^luc^ bladder tumor burden, yet, there were cellular changes in the MB49^luc^ bladder TME following NHS-muIL12 treatment that could represent early events that ultimately would provide an immunopermissive TME leading to MB49^luc^ bladder tumor regression. Those changes include (1) decrease in the number of Ly6C^+^ and Ly6G^+^ MDSCs (Fig. 3a, b), (2) increase in M1 macrophages, as identified by CD38 expression [34] (Fig. 3c) and (3) increase in CD4^+^IFN-γ^+^ cells (Fig. 5d). Those changes would be expected to begin the conversion of the MB49^luc^ bladder TME from one of immunosuppressive to proinflammatory following NHS-muIL12 treatment.

By 144 h after the final NHS-muIL12 treatment, the cellular makeup of the MB49^luc^ bladder TME had dramatically changed with the number of MDSCs and macrophages significantly reduced which, in turn, remodeled the MB49^luc^ bladder TME by increasing the ratios of CD4^+^ and CD8^+^ to CD11b^+^Gr1^+^F4/80^−^ MDSCs and CD11b^+^Gr1^−^F4/80^+^ macrophages (Fig. 2g-j). When MDSCs were further divided into monocytic (CD11b^+^ Gr1^+^ Ly6C^+^ Ly6G^−^) and granulocytic (CD11b^+^ Gr1^+^ Ly6C^low^ Ly6G^+^), NHS-muIL12 treatment kept a low number of monocytic MDSCs while virtually eliminating granulocytic MDSCs within the MB49^luc^ bladder TME (Fig. 3a, b). These data are consistent with a previous finding that a robust increase in intratumoral CD8^+^ T cell activation and proliferation resulted after targeted depletion of granulocytic MDSCs [37]. Those changes within the myeloid cell compartment provide compelling evidence for a reversal of cellular immunosuppression within the MB49^luc^ bladder TME elicited by NHS-muIL12 treatment resulting in significant tumor regression. In addition, the drop in immunosuppressive myeloid cells was also accompanied by an increased response of CD4^+^ and CD8^+^ T cells to exogenous broad-based stimuli (Fig. 4). Thus, phenotypic/functional changes within the MB49^luc^ bladder TME not only affirmed the tumor-targeting ability of NHS-muIL12, but also offered a plausible sequence of events that accompany the significant reductions observed in MB49^luc^ bladder tumor burden. With the initial intravesical inoculation of MB49^luc^ tumor cells, early intratumoral necrosis occurs that releases intrinsic tumor-associated antigens, which, in turn, induces a host immune response. Support for the intrinsically driven host immune response against intravesical MB49^luc^ tumors includes accumulation of immune cells within the tumor mass of control Ig–treated mice [22]. The presence of antitumor immune cells in the MB49^luc^ bladder TME is countered by a large number of immunosuppressive CD11b^+^ Gr1^+^ F4/80^−^ MDSCs and CD11b^+^Gr1^−^F4/80^+^ macrophages. Reduction in the number of MDSCs and macrophages following NHS-muIL12 targeting might also trigger a positive feedback loop wherein local IL-12 production supports IFN-γ accumulation [38]; this reignites the cytolytic abilities of resident CD4^+^ and CD8^+^ T cells leading to the significant regression seen in the MB49^luc^ bladder tumors. Support for this hypothesis includes: (a) in vivo depletion of either CD4^+^ or CD8^+^ T cells abrogates the antitumor effects of NHS-muIL12. In a previous study, MB49^luc^ bladder tumor weights were reduced from 339 ± 61 mg to 137 ± 36 mg in non–T cell depleted NHS-muIL12–treated mice, while no such reductions were evident in NHS-muIL12–treated mice depleted of either CD4^+^ (bladder weights: 298 ± 76 mg) or CD8^+^ T cells (bladder weights: 456 ± 101 mg) [27], (b) regression of the MB49^luc^ bladder tumors occurs at low doses of NHS-muIL12 (i.e., 0.4 μg/injection), and (c) previous results demonstrate that targeting the PD1/PD-L1 axis also mediates regression of the MB49^luc^ bladder tumors [22]. Indeed, successful gene therapy of the MB49 tumors utilizing antigen-specific CD8^+^ T cells engineered to secrete IL-12 has been associated with the promotion of dendritic cell maturation [29, 38, 39]. It is intriguing to point out that in the control Ig–treated mice, in which MB49^luc^ bladder tumor burdens increased during the 72–144-h time interval, CD4^+^ and CD8^+^ effector T cell numbers (Fig. 2d, e) actually increased, yet their activation status fell (Fig. 5a and b), providing additional evidence for immunosuppression. With NHS-muIL12 treatment, the reductions of MB49^luc^ bladder tumor growth was not accompanied by any discernable increase in the number of CD4^+^ and CD8^+^ effector T cells (Fig. 2d, e), yet their activation status was increased (Fig. 5a, b). Those findings argue that the resident T cells were not subject to any intrinsic and/or irreversible defect (e.g., lack of retention, recruitment, exhaustion), but when unshackled from immunosuppressive components they were able to elicit a robust antitumor response directed at the remaining MB49^luc^ bladder tumor cells. Seemingly this switch from an immunosuppressive to an immunopermissive TME is the foundation for the significant antitumor responses observed following NHS-muIL12 treatment. In a recent phase I clinical study in patients diagnosed with metastatic carcinomas [40], the maximum tolerated dose (MTD) for NHS-IL12 was 16.8 μg/kg, clearly within range of doses that elicited significant antitumor effects in the MB49^luc^ non-muscle invasive bladder tumor model. It is intriguing to suggest an additional study of NHS-IL12 in the context of patients diagnosed with carcinoma in situ, and in patients with non-muscle invasive, non-metastatic urothelial carcinoma who are BCG-naïve and/or have not responded to BCG.

Comparing NHS-muIL12 as an immunotherapeutic in the present study with findings from a previous report [18] offers some intriguing differences between treating mice bearing MB49 s.c. versus intravesical (i.e., mucosal) bladder tumors. For instance, the dose range that significantly suppresses the growth of intravesical MB49^luc^ bladder tumors (0.05 to 0.4 μg, ref. [27]) was, for the most part, ineffective when administered to mice bearing MB49 subcutaneous tumors [18]. Secondly, immune cell depletion studies showed that the prime effectors capable of inhibiting MB49 s.c. tumor were CD8^+^ T and NK cells, whereas to effectively reduce MB49 bladder tumors required both CD4^+^ and CD8^+^ T cells. Whether the differences in dosing and effector cells are tied to the underlining differences of tumor within the s.c. space versus the mucosa should be a focus of future studies.

Finally, it should be mentioned that NHS-muIL12 can be easily paired with avelumab, an anti-PD-L1 antibody. In preclinical studies, concurrent administration of these two immunotherapeutics resulted in additive/synergistic antitumor effects in both s.c. transplanted tumors (MC38, MB49) as well as the MB49^luc^ bladder tumor model [41]. It is noted that metastatic bladder cancer, especially in patients treated with prior therapies, can have regions of tumor necrosis, and thus the potential for binding of NHS-IL12 and subsequent anti-tumor activity. Those results informed the design of a phase I clinical trial in which patients diagnosed with metastatic carcinoma were treated with a combination of NHS-IL-12 and avelumab [42].

## Conclusions

NHS-IL12 is a fusion protein comprised of a tumor-targeting IgG associated with IL-12. Administration of NHS-IL12 offers the opportunity to target a potent T_H1_ cytokine to the TME, while mitigating the toxicities associated with systemic administration of recombinant IL-12. In the present study, targeting IL-12 to MB49^luc^ bladder tumors reverts an immunosuppressive to an immunopermissive TME, inducing potent antitumor effects. The findings argue for the continued development of NHS-IL12 as an immunomodulator and potential clinical immunotherapeutic for non-muscle invasive bladder cancer and other cancers.

## Data Availability

The data generated and analyzed will be made available from the corresponding author on reasonable request.

## Abbreviations

Arg I: Arginase I
BCG: Bacillus Calmette-Guerin
CRADA: Cooperative Research and Development Agreement
DPBS: Dulbecco’s phosphate buffered saline
i.p.: intraperitoneal
iNOS: inducible nitric oxide synthase
LAP: Latency-associated peptide
MACS: Magnetic-activated cell sorting
MDSCs: Myeloid-derived suppressor cells
MTD: Maximum tolerated dose
NK: Natural killer
r: recombinant
s.c.: subcutaneously
TME: Tumor microenvironment
